# The implementation of digital biomarkers in the diagnosis, treatment and monitoring of mood disorders: a narrative review

**DOI:** 10.3389/fdgth.2025.1595243

**Published:** 2025-06-17

**Authors:** Andrea P. Garzón-Partida, Citlali B. Padilla-Gómez, Diana Emilia Martínez-Fernández, Joaquín García-Estrada, Sonia Luquin, David Fernández-Quezada

**Affiliations:** ^1^ Departamento de Neurociencias, Instituto de Neurociencias Traslacionales, Centro Universitario de Ciencias de la Salud (CUCS), Universidad de Guadalajara (UdeG), Guadalajara, México; ^2^ Departamento de Farmacobiología, Centro Universitario de Ciencias Exactas e Ingenierías (CUCEI), Universidad de Guadalajara (UdeG), Guadalajara, México

**Keywords:** digital biomarkers, mood disorders, predictive model, machine learning, bipolar disorder, depression, major depressive disorder

## Abstract

Mood Disorders are a group of mental health conditions characterized by a disruption of the emotional state that affects the quality of life of the people living with them. Mental Disorders are difficult to diagnose and treat due to the complex processes involved and limitations of the healthcare system. Digital biomarkers have created accessible, long-term, non-invasive, and user-friendly alternatives for the diagnosis, treatment, and monitoring of these conditions. The use of everyday devices like smartphones and smartwatches and specialized tools like actigraphy, in conjunction with powerful statistical tools, artificial intelligence, and machine learning, represents a promising avenue for the implementation of personalized strategies to monitor and treat Mood Disorders, and potentially higher adherence to treatment. We conducted several studies that implement a variety of methodologies and tools to better understand Mood Disorders, using a patient-focused approach with the ultimate goal of identifying better strategies to improve their quality of life.

## Introduction

Mood disorders (MD) encompass a category of psychiatric conditions characterized by significant disturbances in emotional regulation. These disorders manifest as prolonged or cyclical episodes of depression, hypomania, or mania, impairing daily functioning and overall well-being. The MD spectrum includes Major Depressive Disorder (MDD), Bipolar Disorder (BD), Cyclothymia, Hypomania, Disruptive Mood Dysregulation Disorder, and Premenstrual Dysphoric Disorder (1).

The diagnosis and treatment of MD remain complex due to barriers such as limited access to mental health services and low long-term treatment adherence, often influenced by socio-demographic factors (2). Studies indicate that in developed countries, only 50% of individuals with MD receive diagnoses and follow-up care. This figure drops to 30% globally when developing countries are considered, where stigma surrounding mental health further hinders access to care (3).

## Search method

This article is a narrative review which employed a systematic search process. Articles were collected from previously published original articles in The Web of Science and Pubmed databases, until August 2024. A comprehensive literature search was conducted using a combination of Medical Subject Headings (MeSH) and free-text terms to identify relevant studies examining the use of digital biomarkers in patients with Mood Disorders. The search strategy was designed to capture studies that addressed digital biomarkers in the context of diagnosis, assessment, or monitoring of mood-related conditions. Search terms included combinations of keywords and MeSH terms related to (“digital biomarkers” OR “digital phenotyping” OR “passive sensing” OR “wearable devices” OR “mobile health” OR “mHealth” OR “machine learning”) AND [“mood disorders” [MeSH Terms] OR “depressive disorder” [MeSH Terms] OR “depression” OR “bipolar disorder”] AND (“diagnosis” OR “assessment” OR “monitoring” OR “detection”). These terms were searched within the title, abstract, and indexing fields of each database. Inclusion criteria focused on peer-reviewed original research studies involving human participants, with outcomes relevant to the use of digital biomarkers in mood disorder diagnosis or management. Exclusion criteria included review articles, editorials, conference abstracts, and studies not available in English. Upon full text examination of relevant articles, we included studies that used digital measurements obtained through the use of smartphone devices, wearables or online platforms, on individuals with MD, both depressive and bipolar. After the selection process, data from the studies was compiled on an Excel spreadsheet, coding for variables of interest: authors, year, objectives, MDs, number of participants, duration, dispositive, data type, metrics used and results. Finally, the studies were categorized into categories in accordance to their objective and methodology.

## Clinical and digital biomarkers

Biomarkers are among the most widely used tools for assessing physical and mental health due to their precision and reproducibility (4). Traditionally, psychiatric diagnoses rely on standardized psychological tests (5) and neuroimaging techniques such as electroencephalography (6).

Digital biomarkers, derived from data collected through smart devices, offer an alternative to conventional diagnostic methods (7). These biomarkers provide continuous, longitudinal, and objective metrics of physiological, psychological, behavioral, and environmental variables (8). Wearable technologies, such as smart rings, actigraphy devices, smartwatches, and smart patches, along with smartphones and tablets, leverage the Internet of Things (IoT) to enable seamless data collection and integration. This connectivity allows real-time data transmission through Bluetooth or Wi-Fi, facilitating AI- and ML-driven analysis (9, 10).

Machine learning models (MLMs) process large-scale, complex datasets with high precision, identifying patterns that enhance the understanding, diagnosis, and prediction of symptom severity in psychiatric conditions, including MD (11, 12). These models utilize both passive (automated) and active (user-initiated) data collection, with validation achieved through comparisons with standardized clinical assessments (13, 14).

The assessment of MD relies primarily on self-reported questionnaires and validated clinical scales, including the Patient Health Questionnaire (PHQ-9) for depression, the Generalized Anxiety Disorder (GAD-7) scale for anxiety, and the Young Mania Rating Scale (YMRS) for manic symptoms in BD. Additionally, Ecological Momentary Assessment (EMA) and cognitive performance tests have been widely used for continuous mood and functionality evaluation.

Given the heterogeneity of MD research, studies incorporating digital biomarkers are categorized based on their primary objectives: diagnostic tools, therapeutic interventions, ML-based classification models for symptom monitoring, and predictive analytics for prognosis.

## Digital therapeutic interventions

Clinical trials evaluating digital interventions for MD report mild to favorable outcomes. The predominant approach involves adapting traditional therapeutic strategies, such as cognitive-behavioral therapy (CBT), into self-guided digital formats. Studies examining the efficacy of smartphone-based self-reporting applications (Table 1) indicate overall improvements in depression and anxiety symptoms. For instance, the Vida Health app integrates digital CBT with symptom monitoring, yielding positive results in clinical settings compared to traditional therapy (15). Another study compared standard CBT with blended CBT, assessing its effectiveness through participants' self-reported therapeutic alliance and mood states at a regular basis, resulting on a higher effectivity of blended CBT after mood states were compared between groups (16).

**Table 1:** Therapeutic approaches with the use of digital technologies and digital biomarkers.

Study	Participants	Duration	Dispositive
	*n*	Days	
(19)	456	56	Smartphone; Juli app
(20)	315	30, 60	Smartphone; CORE app
(18)	146	56	Smartphone; app
(15)	323	270	Smartphone; Vida Health Therapy app
(17)	222	117	Smartphones; LiveWell app
(16)	943	90	Computer and smartphone; app
(21)	49	180	Web-based platform Mood FX
(24)	241	90	Web-based platform Life Flex
(23)	680	90	Web-based platform Step-by-Step
(22)	993	112	Web-based platform Dario Health

Additional intervention aimed to teach self-management techniques through specialized activities and self-reports, complementing standard therapeutic approaches. Participants recorded the severity of their symptoms and mood states while receiving coaching on self-management strategies through the LiveWell and IntelliCare apps (17, 18). Also, the Juli app collected passive smartphone data alongside self-reported symptom severity, mood fluctuations, and quality of life indicators, all of which showed improvement following the study (19). A fully remote, app-based intervention tool called CORE demonstrated significant therapeutic effectiveness across participant groups at both the 30-day and 60-day marks, with a 50% adherence rate (20).

Two web-based platforms were evaluated for their usability and acceptability among individuals with depression. MoodFX, a therapeutic tool that also functioned as a tracker for cognitive function, symptom severity, quality of life, medication adherence, and general health status, exhibited high usability, with participants reporting notable improvements in depressive symptoms (21). The Dario Health platform, which incorporated guided coaching interactions for the management of depression and anxiety, demonstrated a reduction in mood disorder symptoms based on user-reported effectiveness scores (22).

The Step-by-Step platform was tested as an intervention tool during Lebanon's economic, environmental, political, and humanitarian crisis amidst the COVID-19 pandemic. Notably, it reported a higher treatment response rate, 1 in 4 participants, compared to traditional therapy, which had a response rate of 1 in 7, even at the three-month follow-up (23). Life Flex, designed for individuals with MDD and BD, was effective in reducing anxiety, depression, psychological distress, and emotional dysregulation. The intervention was well-accepted by users, with adherence rates extending up to 90 days (20).

## Correlation models

The Correlation models are statistical techniques used to analyze linear relationships between variables, even when they appear dissimilar (25). Several studies have explored correlations between digital biomarkers, such as physical activity levels, sleep patterns, geolocation data, and speech markers, derived from wearable devices and self-reported clinical scales measuring symptom severity. Study protocols varied in duration, with those lasting ≤14 days classified as short-term and those exceeding 14 days categorized as long-term.

## Exploratory short-term studies

Short-term correlation studies (Table 2) included medium-to-large participant samples (n) observed over brief periods. MLMs processed extensive datasets obtained from wearable devices and specialized applications to generate digital profiles of participants. Despite methodological diversity across studies, all utilized smartphone-based data collection.

**Table 2:** Correlations between digital data and self-report scores for diagnosis in short-term studies.

Study	Participants	Duration	Dispositive
	*n*	days	
(15)	286	14	Accelerometer; smartphone
(17)	242	14	Actigraph; smartphone
(16)	87	>4	Smartphone; smart patchers; Mood Zoom MZ app
(21)	74	14	Actigraph; smartwatch
(24)	77	1	Smart glasses Emteq's ECOsense™
(22, 23)	84; 290	14; 14	Smartphone; personalized app
(25)	1,423	14	Smartphone; app Juli

In studies employing secondary devices, actigraphs, smartwatches, smart patches, and smart glasses were utilized. The DynaPort Hybrid accelerometer was used to assess gait characteristics, including abnormalities and movement patterns, correlating these features with depression scores from two databases of adult and elderly participants. The results revealed a negative correlation between regular gait behavior and depression scores, allowing for a passive assessment through regular behavior (26). Similarly, the research-grade Actigraph Motion Watch 8 and the commercial-grade Galaxy Watch Active 2 were employed to develop a depression phenotype by measuring psychomotor retardation and agitation, with variations correlating to different depressive episode presentations regardless of the dispositive used for measurements (27).

Physiological measurements were collected using the Philips Respironics Actigraph and Proteus smart patches, which detect electrodermal activity, physical activity (PA), and sleep patterns. These studies identified associations between PA levels, energy expenditure, and activity duration with the mood states of participants with BD (28). Additionally, integrating the Proteus patch with the Mood Zoom app allowed researchers to analyze heart rate (HR), PA, sleep, and circadian rhythms, establishing a link between diurnal activity patterns and mood fluctuations in participants with BD and BPD (29).

Specialized wearables, such as Emteq's ECOsense™ smart glasses, demonstrated the versatility of digital biomarkers. Using optomyography-based sensor data, a one-session protocol assessed emotional states in participants with depression, comparing them to healthy controls (HCs) and self-reported measurements of arousal and valence after exposure to visual stimuli. Results indicated a correlation between muscle activation and self-reported emotional scores, distinguishing depressed participants from HCs based on facial muscle response intensity during emotional stimuli (30).

Smartphone-based studies utilized both passive and active data collection methods to characterize participant profiles. One study implemented a mobile app that leveraged Global Positioning System (GPS) data to assess mobility, activity at specific locations, and travel patterns, correlating these behaviors with depressive symptoms, confirming a negative correlation between lowered physical activity and a higher depression scores (31). Additionally, GPS data was used to determine participants' approximate geographical locations, facilitating the acquisition of air quality index (AQI) ratings and weather conditions throughout the day, particularly the approximated air pollution of the areas visited, based on the levels reported by meteorological services. This study found a significant relationship between AQI levels and mood disorder symptoms in individuals with BD, with a one-point increase in AQI correlating with a 0.11-point rise in depression severity scores, suggesting a potential link between air pollution exposure and worsening mood symptoms (32).

Within the same category of short-term monitoring, researchers analyzed passive smartphone-recorded social interactions in individuals with depression, individuals with anxiety disorders, and healthy controls. The collected auditory data, including speech characteristics and environmental noise, was used to examine correlations with depression and anxiety severity, individual functionality, and physiological measures such as PA and sleep patterns. The analysis revealed a negative correlation between social interactions and depression severity, though no significant relationship was observed for anxiety symptoms (33). These findings reinforce the potential of alternative digital biomarkers as valuable tools for monitoring and treating mood disorders, further validating the well-established relationship between reduced social interactions and increased depressive symptoms (34).

## Exploratory long-term studies

In the second category (Table 3), monitoring durations ranged from 89 to 391 days, with the largest variation in sample sizes (n), ranging from 30 to 10,718 participants. Digital sensor-based and self-reported measurements were used to monitor participants in real-world environments over extended periods, allowing for real-time data collection and deeper insights into the lived experiences of individuals with mood disorders (35).

**Table 3:** Correlation models through long-term monitoring of symptoms in MD.

Study	Participants	Duration	Dispositive
	*n*	days	
(32)	30	42	Smartwatch; smartphone
(33)	40	56	Actigraph
(34)	139	391	Smartwatch; smartphone
(35)	66	336	Smartwatch; Ecological Momentary Cognitive Testing app
(36)	163	292	Tablet; VoiceSense app
(37)	316	266	Smartphone; Monsenso app
(38)	173	28	Smartphone; ReMAP app
(31)	10,718	84	Online platform Talkspace

Actigraphy and smartwatches have proven to be viable tools for collecting long-term data, with study protocols extending up to 336 days. In one study, circadian rhythms, mood fluctuations, and symptoms in participants with mood disorders were monitored using a combination of Fitbit Charge HR 2 and Fitbit Charge HR 3, smartphone-delivered self-assessment EMA, and standardized clinical scales. Results demonstrated a discernible relationship between circadian phase disturbances, mood fluctuations, and symptom severity in individuals with BD, highlighting circadian rhythms as potential variables for digital phenotyping of BD mood state (36). Similarly, the effect of circadian rhythms on individuals with MDD was investigated using the ActiCal actigraph measurements of PA, analyzed for variability. This study examined medication effects in both remitters and non-remitters, revealing a significant correlation between symptom remission and circadian rhythm stability, allowing for a potential use of circadian rhythm stability as a potential biomarker for treatment effectivity (37).

In addition to passive data collection and self-assessments, cognitive function was monitored in participants with BD and MDD using smartwatches. A study integrating Apple Watch physiological data, smartphone-delivered cognitive tests, and self-reported symptom tracking evaluated long-term changes in mood, cognition, PA, and HR in participants with MDD. Findings indicated a correlation between mood fluctuations, PA levels, and HR, as well as a positive relationship between increased PA and improved mood (38). Another study assessed cognition and symptom fluctuations in individuals with BD using smartwatch-delivered EMA, while also evaluating usability and engagement with the LiveWell self-management app. Results showed a significant impact of cognitive performance on depression and mania scores (39). Showing a viability of cognitive performance assessment in non-clinical settings for a greater insight into variability, emotional state, and environmental factors.

Furthermore, PA and stress levels were monitored through passive data collected via the Monsenso smartphone app in a study assessing mood stability in individuals with BD. Results indicated that greater mood stability was positively correlated with stable PA levels and perceived stress regulation, while negatively correlated with quality-of-life indicators. This data was collected through daily self-reports for up to 266 days, (40). A similar relationship was observed in another 28 days-long study examining individuals with MDs and anxiety disorders, where symptoms and mood stability were measured through passive data and self-reports using the ReMAP app, which was consistent with clinical scales of depression and anxiety, accounting for effectiveness in shorter periods of times (41).

Language and speech patterns have also been utilized in long-term correlation models through at least two distinct approaches. The first approach involved analyzing acoustic content-free speech features and behavioral trends via the VoiceSense app to classify speech patterns and vocal features indicative of depression. This method was particularly effective in individuals with high PHQ-9 depression scores, with results also highlighting the influence of age and educational level on speech characteristics (42). The second approach involved analyzing the content of text-based therapy sessions from the Talkspace web-based therapy platform. In this study, behavioral activation words, Linguistic Inquiry and Word Count (LIWC) markers, and time-related metadata from interactions were successfully correlated with established depression markers and clinical scores, allowing for an assessment of therapy efficacy and depression improvement through time (43).

## Classification models of MD diagnosis

MLMs can be implemented using various algorithms, each designed for specific tasks based on the type of data and the underlying model architecture. Among the two primary types of MLMs, supervised and unsupervised, the reviewed literature primarily focused on supervised models, which were used to classify data, establish regressions, or perform both tasks simultaneously (44).

For studies employing classification models, data were categorized to identify and group information into predefined categories (45). By recognizing patterns, these models allowed diverse datasets to be assigned to specific classes, effectively distinguishing between HCs and individuals with MDs, as well as identifying disorder-specific characteristics (Table 4).

**Table 4:** Classification MLMs of MD diagnosis.

Study	Participants	Duration	Dispositive
	*n*	days	
(42)	75	14	Smartband
(43)	139	84	Smartband; smartphone; mindLAMP app
(44)	55	14	Actigraph
(45)	31	56	Smartband; smartphone; personalized app
(46)	140	2	Smartband EDA
(47)	138	180	Smartwatch; smartphone; Monsenso system app
(48)	59	7	Activity monitor Open Lab: Open Movement
(49)	87	28	Actigraph; smartphone; Mood Zoom app
(50)	69	2	EDA wristband
(51)	22	21	EDA device; online screening tool
(52, 53)	40; 79	14; 360	Smartphone; personalized app
(54)	36	60.41	Smartphone; True Colors app
(55)	39	450	Smartphone; BiAffect app
(56)	54	30	Smartphone; smart ring Oura; app
(57)	105	6	Smartphone; mEMA app
(58)	104	28	Smartphone; Sonde Health app; web-based platform SurveyLex
(59)	89	1	Tablet; headset; microphone
(60, 61)	78; 70	1; 2	Web-based platform

Wearable devices have proven instrumental in providing data to train MLMs and develop phenotypic classifications for MDs. The Actiwatch actigraph, for instance, successfully detected changes in depressive symptoms, enabling the diagnosis of participants with MDD and BD by identifying movement patterns and activity characteristics (46). Similarly, the GENEActiv Original actigraph was used to examine the variability of circadian rhythms, correlating mood instability and symptom severity with sleep variability, which effectively distinguished between BD, BPD, and HCs (47). The validated precision of these devices further strengthens PA and circadian rhythm as reliable biomarkers in the assessment of MDs and symptoms intensity.

Smartbands with different levels of precision have also been employed for this purpose, with the Empatica E4 collecting physiological data later used to train deep-learning models and MLMs that outperformed traditional psychological scales such as the Hamilton Depression Rating Scale (HDRS) and the YMRS (48). Additionally, Fitbit Charge HR 2 and HR 3 were used to obtain physiological and behavioral data, which, through MLM analysis, successfully correlated circadian disturbances with MDD and BD-I, leading to the development of a diagnostic models for both conditions, where a personalized model outperformed a generalized one by 23.8% accuracy (49). A less commonly utilized wearable, the Oura smart ring, which integrates mobile usage data and GPS capabilities, was employed in a study where participant mobility patterns, including the duration and type of locations visited, along with sleep characteristics, such as sleep onset and daily variability, served as reliable markers for accurately classifying depression through multi-level models. Combined models where data from all devices and self-report measures, obtained the best predictive performance (50).

Another key physiological measure used in MLM-based classification models was electrodermal activity (EDA). Three separate studies explored EDA variations among participants with MDD, BD, and HCs, aiming to classify mood states and symptoms for diagnostic purposes. In individuals with BD, the Empatica E4 detected mood-related EDA variations, with peaks per minute correlating negatively with depressive episodes and positively with manic episodes, thereby distinguishing between BD participants' emotional states (51). Additionally, another study utilizing the same device determined that wakefulness EDA was the most reliable classifier for differentiating participant groups, with symptoms, body temperature, and medication use emerging as the primary variables influencing the model. Nightly EDA. All MLM, regardless of the circadian phase, were significantly affected by mixed symptoms, skin temperature, anticholinergic medications. Sex was significant for tonic phases of sleep, while age didn't affect any model (52). The ADI Study Watch, which functioned as both a physiological monitoring tool and a digital symptom diary, detected significant positive correlations between increased skin conductance responses and depressive symptoms such as anhedonia, guilt, sleep disturbances, and appetite changes. Centrality predictive strength was found with a higher intensity for suicide, followed by skin conductance and depressive moods. These findings contributed to the development of a model capable of accurately identifying individuals with MDD (53).

## Smartphone-based diagnostic MLMs for MDs

Smartphone GPS data and specialized mobile applications have been leveraged to develop diagnostic MLMs for BD. One study distinguished BD participant from HCs by analyzing variations in location entropy and mobility patterns. GPS data with PA and sleep parameters were integrated, enabling the detection of BD and classification of symptom states. The highest accuracy (F1 = 0.86) was achieved for the detection of depressive symptoms (54). Furthermore, an MLM integrating EMA data successfully distinguished BD, schizophrenia, and schizoaffective disorder, revealing small to moderate correlations between participant location data and negative symptoms, functional outcomes, and active behaviors, while positive symptoms, depression, and anxiety exhibited lower correlations (55).

MLMs have also been applied in the multidimensional assessment of cognition in MDs. One study combined Magnetic Resonance Imaging (MRI), motor characteristics, PA monitoring using the Open Lab: Open Movement 1, memory and attention tests, and standardized depression scales to examine late-life depression. Results revealed significant associations between late-life depression and slower PA, decreased fine motor skills, impaired learning, and reduced quality of life, leading to the development of a classification tool based on depression severity scores (56, 57). Another study employed the Fitbit Ionic smartwatch alongside the Monsenso system to monitor cognition, PA, sleep, and geolocation, enabling the identification of depressive, manic, and euthymic states in BD participants. Notably, the frequency of phone calls per day was found to correlate significantly with mood states. While significant correlations were found, the MLM couldn't be validated (AUC = 0.42) (40). Similarly, an MLM analyzing smartphone-based socialization patterns and cognitive assessment activities successfully diagnosed depression using standard clinical outcome measures, achieving an accuracy of 80%–85% when the NeuroCart battery for eye movement was used as classifier (57).

One study utilized multimedia data in diagnostic MLMs, incorporating headsets and cameras to analyze facial and vocal features for distinguishing MDs from other psychiatric disorders. The study found that muscle and voice activity variations could discriminate between schizophrenia and BD while also quantifying symptom severity, achieving a performance up to AUROC = 0.81 in the prediction of the symptom of blunted affect by voice features, and down to AUROC = 0.61 for worthlessness (58). The Sonde Health smartphone app, complemented by self-assessment data, developed a model capable of stratifying individuals by mental health risk, further refining classification accuracy by integrating self-reported symptomatology and protocol completion rates. Accuracy of assessment was dependent of the level of engagement with the App, with a 38% of participants achieving the best predictor level (high) (59). Additionally, the BiAffect smartphone app successfully detected depression severity changes through typing behavior analysis, with key parameters including typing speed, variability, accuracy, session duration, autocorrect usage, and overall device interaction patterns. Age, typing session duration and one or two-hand typing were factors that affected the typing speed, variability, use of autocorrect and pauses (60).

A high-performance MLM was developed by integrating multisource data from the Empatica E4 wristband, a smartphone, and a specialized mobile application. Monitoring was carried out using sensors on both hands, with a variance of daily data from 17 to 15.5 h. This model incorporated self-reported symptom assessments, EDA, sleep patterns, motion data, location tracking, and social interaction metrics (e.g., phone calls and SMS frequency), demonstrating superior classification accuracy for mood disorder diagnosis (61).

## Web-based platforms for MD diagnosis

While most studies reviewed in this section relied on wearables and smartphones, two investigations utilized web-based platforms. The first study compared MDD symptomatology with subthreshold depressive symptoms using digital assessments. The platform effectively distinguished between clinical MDD and subthreshold depression, improving classification accuracy through transdiagnostic symptom analysis. However, results indicated a tendency toward overdiagnosis in PHQ-9-based assessments (62). Another study applied an Internet-Based Cognitive Assessment Tool (ICAT) to evaluate cognitive function in BD, successfully linking cognitive impairments with poorer functional outcomes and symptom severity (63).

## Predictive models of MD prognosis

Predictive models utilize mathematical and computational techniques to estimate the likelihood of positive, negative, or stable clinical outcomes based on behavioral, physiological, and environmental data (64, 65). As outlined in Table 5, these models have been employed to predict remission and relapse rates, symptom severity trajectories, and cognitive functioning changes across varying timeframes.

**Table 5:** Predictive models of MD prognosis.

Study	Participants	Duration	Dispositive
	*n*	Days	
(81)	*183*	7	Actigraph, smartphone; D-MOMO app
(74)	19	56	Smartwatch, smartphone; app
(66)	68	56	Actigraph
(67)	271	≥365	Actigraph, smartphone
(80)	*55*	30	Smart ring, smartphone, Delphi app
(72)	*270*	91	Smartwatch, smartphone, eMoodChart app
(78)	*252*	56	Smartphone, MindLAMP app
(77)	29	365	Smartphone, MovisensXS and ChronoRecord system apps
(68, 70, 71)	84; 159; 59	7; 94.25; 133	Smartphone; personalized app
(69)	31	7	Smartphone; Mood Triggers app
(76)	65	56	Smartphone; HelloBetter, MAIKI and Insight apps
(73)	*437*	280	Smartphone, AWARE framework app
(49)	*629*	22.1	Smartphone; Carat app
(82)	*316*	14	Smartphone; RADAR-base pRMT app
(75)	18	56	Smartphone; BiAffect app
(79)	334	84	Smartwatch; smartphone; Mood Mirror app
(83)	60	77.08	Online platform True Colors
(84)	*683*	2,190	Online platform Facebook

Chronobiological data obtained from Fitbit smartwatches, smartphones, and a specialized app successfully predicted three-day mood states with 65% accuracy for manic and hypomanic episodes, particularly for a personalized model, which outperformed the general by 23.8% (66). A dynamic network analysis of PA, measured via Actiwatch actigraphs and clinical assessments, predicted worsening depressive symptoms in BD-I, with declines in PA serving as an early indicator. Interestingly, the dynamic network structures were constructed using multilevel vector autoregressive (VAR) analysis, where each affect item and physical activity feature was classified as a node (67).

Furthermore, by implementing activity anomaly detection frameworks (N-of-1) algorithms, one study predicted MDD relapse with ≥71% accuracy, up to 2–3 weeks in advance, from data of patients from two different cohorts of people with and without at least one relapse (68). Another study utilized sleep data and EMA self-assessments to create a one-day mood forecast MLM, in which past mood was identified as the most accurate and least error-prone predictor. Lineal and ordinal hierarchical models were evaluated, finding lineal models to be better at catching distribution tails, and the Bayesian ordinal model better at data grouping and predicting mood levels (69).

Patterns of movement, tracked using smartphone-integrated GPS, were incorporated into predictive MLMs. The Mood Triggers app monitored symptoms and correlated them with PA and pre-defined locations, enabling the creation of a model that predicted the onset of depressed moods in individuals with MDD up to one hour before onset (70). Additionally, self-reported sleep quality, mood, and passively collected PA data were identified as reliable predictors of next-week mood and symptom outcomes in BD participants. In this study, data was approached through distinct time frames: repeated, daily, weekly and monthly, allowing a deeper correlation analysis (71). Another MLM achieved higher precision using a group-personalized regression model, correlating diurnal movement patterns, transition time between locations, total traveled distance, variability of location, and time spent at home with symptom severity in BD (72).

MLMs for predicting mood episodes were developed using circadian rhythm data. Passively collected daily activity patterns, obtained from a Fitbit Charge HR smartwatch, a smartphone app, and self-reported mood assessments, contributed to models that accurately predicted depressive, manic, and hypomanic episodes by linking low sleep quality, abnormal PA, and circadian disruptions with an increased likelihood of manic or hypomanic episodes (73). Similarly, a high-accuracy predictive model of depressive symptom severity, capable of forecasting symptoms up to three weeks in advance, was developed using circadian rhythm data, socialization patterns, and digital behavior collected via the AWARE framework smartphone app (74).

Predictive typing behavior-centered MLMs were developed by analyzing typing speed, accuracy, correction patterns, and autocorrect use, combined with EMA-derived mood and cognitive performance data. Typing speed was identified as a predictor of attention and processing skills, correlating with depressive symptoms. Three mixed longitudinal MLMs were implemented, with all of them accounting for age and time of the day, and a possible practice effect on keystroke behavior (75). The BiAffect app further demonstrated predictive capabilities by analyzing time, quality, and duration of typing sessions, successfully forecasting depression scores in BD participants (76).

Smartphone usability features have been explored as potential markers of communication behaviors and digital activity. One study using the Carat app monitored screen time and app usage, identifying positive correlations between screen activity patterns and depressive symptoms. MLMs trained on these data successfully predicted depression severity based on screen and internet connectivity metrics (50). Additionally, complex models integrating metadata from the HelloBetter app, symptom self-assessments via the MAIKI app, and phone usage data from the Insight app generated an MLM capable of assessing depression severity. However, this model exhibited a notable sex-based bias, overrepresenting female participants (77). Similarly, correlating passive smartphone PA and sleep data (collected via the MovisensXS app) with phone activity data from the ChronoRecord system apps, allowed for the successful prediction of same-day manic and depressive episodes in BD participants (78).

Multimodal data collection, incorporating multimedia records, has been employed to predict mania and depression severity in BD patients, testing seven MLMs. The most effective at predicting were the Least Absolute Shrinkage And Selection Operator (LASSO) and ElasticNet regularization moels, which were improved through a greater combination of heterogeneous data (79). By integrating phone and app usage data, MLMs were able to forecast mood variability in BD, with step count and sleep patterns emerging as the most accurate predictive metrics, whereas app usage data exhibited lower predictive accuracy. In all combinations, Random Forest models outperformed others (80). When data from the Oura smart ring was combined with phone usage data, multilevel MLMs achieved higher accuracy in predicting depression and anxiety symptom severity in participants with MDD (81). Further, physiological data, such as heart rate (HR) collected using multiple actigraphs in the same protocol with the D-MOMO app, was found to be a unimodal predictor of MDD, while integrating facial expression analysis, vocal recordings, and self-reported mood significantly improved MLM performance (82).

A multimodal approach has also been employed to assess socialization patterns. By passively collecting Bluetooth-based proximity data from smartphones, researchers calculated socialization time and interaction levels. MLMs successfully correlated reductions in social interactions with worsening depressive symptoms, highlighting the role of circadian rhythms, social engagement, and location frequency in mood prediction (83).

Finally, online-based platforms have been utilized for training, validating, and verifying predictive models. Longitudinal data collected via the True Colors platform established a link between insomnia and mood instability, influenced by demographic and clinical factors. The weekly inertia of sleep quality emerged as a predictor of hypomanic and depressive symptoms in BD (84). Additionally, an analysis of Facebook posts spanning six years was used to train MLMs capable of predicting future depression onset using linguistic markers. The models identified seven out of ten language topics previously associated with depression as predictors of future depressive episodes (85).

## Limitations of digital biomarkers

The use of digital biomarkers has expanded in both research and clinical settings as diagnostic, therapeutic, and disease-monitoring tools for individuals with physical and mental health conditions. These biomarkers also offer enhanced self-management opportunities for patients (86). However, as their implementation grows, several methodological, technological, and ethical challenges have emerged.

A key methodological limitation is heterogeneity in study protocols. Luo et al. (87) proposed standardized guidelines for developing MLMs in biomedical research, highlighting the challenge of determining optimal data collection durations. Study lengths have ranged from less than 30 min (60) to two years (65), raising concerns about underfitting or overfitting when developing statistical and machine learning models.

Another critical issue is sample size selection. Rykov et al. (88) proposed a formula for estimating appropriate sample sizes in biomedical research. While some researchers advocate for larger sample sizes to enhance model accuracy (89), others suggest that smaller, high-quality datasets may improve clinical applicability and reduce computational biases (90).

Additionally, sensor accuracy and feature detection reliability have been scrutinized (91, 92). Given the variability in sensor quality, especially between commercial and research-grade devices (93), there is a growing demand for greater transparency regarding measurement accuracy and participant characteristics (95). Validation efforts should focus on comparing digital biomarkers against established clinical measures and standardizing data reporting across studies (93).

Ethical considerations surrounding privacy and data security have also been raised. Given the sensitive nature of digital health data, concerns have emerged about data collection, storage, and processing practices. A great concern regarding privacy and informed consent has been addressed, particularly regarding understanding the true magnitude of the data collected through digital tools, within age and cognitive performance groups, particularly with elderly participants and in cases where participants present a condition where cognition can be affected (96).

While international regulatory bodies have established privacy guidelines for the private sector (97), biomedical research regulations for digital biomarkers remain underdeveloped, necessitating clearer ethical frameworks (94, 95).

Finally, the widely discussed bias present in both digital sensors and algorithms, can be a limitation of digital health technologies applied to mental health. This ethical concern, in addition to the existing race and sex-related bias in the biomedical research, can potentially represent a risk of use of these technologies in underrepresented groups (98).

## Conclusion

The integration of digital measurements, whether obtained through specialized sensors, passively collected metadata, or user interactions with technology, is rapidly transforming mental health research and clinical practice. These emerging methodologies offer non-invasive, cost-effective, and scalable solutions for the diagnosis, monitoring, and treatment of MDs, ultimately improving patients' quality of life.

Studies investigating digital biomarkers for MDs have demonstrated promising results, reducing clinician bias by moving beyond static, clinical assessments and incorporating real-world, dynamic data. However, these technologies also present methodological, accuracy-related, and ethical challenges that must be addressed to ensure their widespread acceptance and clinical validity.

Future perspectives in the field of digital health would benefit by an emphasis on the standardization of the digital tools used in studies, providing a better understanding of the accuracy of the tools and sensors used in the measurement of the metrics. Transparency on the methodology used for validation of datasets, disclosure of the quality of data and its processing could also strengthen possible weaknesses originated by the variability of participants' behaviors in highly heterogeneous environments.

Study protocols in this area can greatly benefit from an interdisciplinary approach. This is by the collaboration between healthcare and mental health professionals, as well as data and biotechnology professionals, to ensure better rigor through the research process.
